# Draft Genome Sequence of Weizmannia coagulans MB BCM9, a Stable Spore-Forming Probiotic

**DOI:** 10.1128/mra.01212-22

**Published:** 2023-02-02

**Authors:** Gaurav Kaushik, Mruduka Patel

**Affiliations:** a Meteoric Biopharmaceuticals Pvt. Ltd, Ahmedabad, Gujarat, India.; University of Rochester School of Medicine and Dentistry

## Abstract

Weizmannia coagulans MB BCM9 (MTCC 25157) is a safe probiotic strain. Here, we announce a fully assembled draft genome sequence consisting of 3,450,803 bp, with 139 contigs. A total of 3,377 protein-coding genes, 15 rRNAs, 80 tRNAs, 5 noncoding RNAs (ncRNAs), and 107 pseudogenes were identified from this assembly.

## ANNOUNCEMENT

Probiotics are commonly defined as live microorganisms that, when administered in adequate amounts, confer a health benefit on the host (1).

Weizmannia coagulans MB BCM9, a spore-forming lactic acid bacterium, has long been considered safe and suitable for consumption. Weizmannia coagulans MB BCM9 is a probiotic with great potential which has been used to relieve symptoms of irritable bowel disease (2), ulcerative colitis, Crohn’s disease, or stomach ulcer associated with Helicobacter pylori infection (3), enhance immunity (4), inhibit the growth of pathogenic bacteria (5), and treat diarrheal diseases in the human body (6). The functional attributes of a probiotic strain are representations of the genome of the bacteria.

Weizmannia coagulans MB BCM9, was preserved in the Microbial Type Culture Collection and Gene Bank (MTCC) as MTCC 25157. The strain was cultivated in a Bacillus coagulans agar containing glucose yeast extract at 40 ± 2°C for 72 ± 4 h. The broth was centrifuged, and the resulting pellet was used for DNA extraction using the XcelGen bacterial genomic DNA (gDNA) isolation kit. The DNA quality and concentration were examined using an 0.8% agarose gel and the Qubit 2.0 fluorometer, respectively. The isolated DNA was amplified using 16S rRNA specific primers (8F and 1401R) and enzymatically purified. It was then bidirectionally sequenced on an ABI 3730xl genetic analyzer. The consensus 16S rRNA gene sequence generated was used for a BLAST search against the NCBI GenBank database, which showed the best hit against strain LBSC (GenBank accession no. CP022701.1). The extracted DNA was used for whole-genome sequencing library preparation using the TruSeq Nano DNA library prep kit as per the manufacturer’s instructions. The quality of the library was checked using the Bioanalyzer 2100 system (Agilent Technologies) with a high-sensitivity (HS) DNA chip, and the library was sequenced with 2 × 150-bp chemistry on the Illumina HiSeq 2500 platform. A total of 7,427,126 paired-end (PE) reads, equivalent to 2.19 Gb, were generated at Xcelris Labs Ltd. (Gujarat, India). The quality of these data was checked using FastQC v11.8 (7), and the adaptors were trimmed using Trimmomatic v0.36 (8).

*De novo* assembly of the high-quality PE reads of MB BCM9 was accomplished using Velvet v1.2.10 (9), and the assembly was optimized at kmer 107. Gaps in the scaffolds were further filled with the PE reads using Gap Closer v1.12 (10). The refined assembly obtained was considered the final assembly for further processing. A total of 7,427,126 paired-end reads were assembled into 139 scaffolds (*N*_50_, 54,331 bp), with the largest assembled contig 197,114 bp long. The draft genome consists of 3,450,416 bp with a GC content of 46.37%. The 139 assembled scaffolds were submitted for PGAP analysis, and as a result, a total of 3,377 genes were predicted; among these, 3,277 coding sequences (CDSs), 15 rRNAs, 80 tRNAs, 5 noncoding RNAs (ncRNAs), and 107 pseudogenes were identified. The genome also contains 1 CRISPR array. Default parameters were used for all software. Of the predicted genes, 392 proteins were identified that are involved in central carbohydrate metabolism, including the glycolysis, pentose phosphate, and xylose utilization pathways (11). Genes related to dormancy and sporulation were annotated, in particular, spore DNA protection/spore coat, the sporulation mechanism, and spore germination. Furthermore, the genome contains determinants involved in the adhesion (i.e., fibronectin- and mucus-binding proteins) and active metabolism in the host (as a biotin biosynthesis pathway).

The complete genome sequence of MB BCM9 (MTCC 25157) obtained in the present study will provide deeper insight into the safety features of this strain, and comparative genomic analysis with the genomes of other *Bacillus* strains might shed new light on the molecular mechanisms at the basis of its probiotic and beneficial properties.

### Data availability.

This whole-genome shotgun project has been deposited at NCBI/GenBank under the accession no. JABDSL000000000. The species identification sequence is available at GenBank under the accession no. MN736120. The raw sequence reads have been deposited in the NCBI Sequence Read Archive (SRA) under the BioProject accession no. PRJNA629465 and the BioSample accession no. SAMN14776668.
